# Emerging Therapeutic RNAs for the Targeting of Cancer Associated Fibroblasts

**DOI:** 10.3390/cancers12061365

**Published:** 2020-05-26

**Authors:** Laura Santana-Viera, Maria L. Ibba, Deborah Rotoli, Silvia Catuogno, Carla L. Esposito

**Affiliations:** Institute Experimental Endocrinology and Oncology “Gaetano Salvatore” (IEOS), National Research Council (CNR), 80145 Naples, Italy; laura_sv@icloud.com (L.S.-V.); m.ibba@studenti.unina.it (M.L.I.); deborah.rotoli@ieos.cnr.it (D.R.)

**Keywords:** tumor microenvironment, cancer associated fibroblasts, therapeutic RNAs

## Abstract

Tumor mass consists of a complex ensemble of malignant cancer cells and a wide variety of resident and infiltrating cells, secreted factors, and extracellular matrix proteins that are referred as tumor microenvironment (TME). Cancer associated fibroblasts (CAFs) are key TME components that support tumor growth, generating a physical barrier against drugs and immune infiltration, and contributing to regulate malignant progression. Thus, it is largely accepted that therapeutic approaches aimed at hampering the interactions between tumor cells and CAFs can enhance the effectiveness of anti-cancer treatments. In this view, nucleic acid therapeutics have emerged as promising molecules. Here, we summarize recent knowledge about their role in the regulation of CAF transformation and tumor-promoting functions, highlighting their therapeutic utility and challenges.

## 1. Introduction

A growing body of literature is demonstrating that cancer progression is a multi-factorial process to which the transformation of the tumor cells contributes only in part. Indeed, the presence of a complex tumor microenvironment (TME) plays a crucial role in tumorigenesis and metastasis and tumor resistance to therapeutic interventions [1,2,3]. Key components of TME are cancer associated fibroblasts (CAFs). Differently from normal fibroblasts (NFs), CAFs are proliferative and metabolically active cells with a myofibroblastic phenotype characterized by the overexpression of some biomarkers, such as α-smooth muscle actin (α-SMA) and fibroblast activation protein (FAP) [4]. It is believed that CAFs actively regulate malignant progression and drug resistance through the secretion of various tumor growth factors, cytokines and matrix remodeling proteins and the induction of the epithelial–mesenchymal transition (EMT) [5,6]. Even if a complete understanding of the molecular mechanisms involved in CAF transformation and functions is still lacking, it is evident that effective anti-cancer strategies should address CAF targeting.

Therefore, there are growing efforts to characterize gene pathways and epigenetic networks implicated in CAF phenotype in order to identify key targetable proteins and RNAs that can allow the development of new precise and effective therapeutics. In this view, endogenous non-coding RNAs (ncRNAs) and synthetic oligonucleotides (ONs), including microRNA (miR)-based molecules, long ncRNAs (lncRNAs), small interfering RNAs (siRNAs), antisense oligonucleotides (ASOs), mRNA therapeutics, and nucleic acid aptamers, offer effective and specific therapeutic tools to regulate the expression or the function of target proteins by distinct mechanisms. These molecules show unique advantages compared to conventional therapies, being endowed with high target-specificity and allowing drugs to address previously intractable targets [7]. NcRNAs are a large group of molecules that comprises, among the most studied, short miRs and siRNAs. These molecules are able to inhibit gene expression at a post transcriptional level through the interference RNA (iRNA) process, either blocking protein translation or causing target mRNA degradation. IRNAs play a crucial role in several pathologies, including cancer, and in the last years they appear to be causally involved in CAF transformation and in the regulation of their tumor promoting effects. Moreover, it has been demonstrated that CAFs can influence cancer cells by releasing miRs into the tumor environment [8]. These studies suggest that mi/siRNAs represent “ideal” therapeutic tools for TME targeting. In addition, short therapeutic ASOs can be also used to degrade RNAs over-expressed in CAFs, thus interfering with their phenotype and pro-tumor functions. Further, endogenous lncRNAs are recently emerging as mediators of CAF communication with cancer cells, and are attracting a great interest as potential new drug targets [9]. In recent years, the use of exogenous mRNAs for therapeutic purposes has also had a great expansion thanks to accumulated knowledge on nucleic acid chemistry and the reduction in production costs, as well as the rapid progression and development of proper delivery methods [10,11,12]. MRNA-based therapeutics can be used for the treatment of different disorders, including cancer, and can be potentially developed also to interfere with CAFs phenotype and pro-tumor functions. Differently from other RNA-based drugs, nucleic acid aptamers are synthetic short single-strand oligonucleotides that bind their target forming 3D globular structures [13]. These molecules are high affinity ligands and potential antagonists of disease-associated proteins with many advantages [14]. Despite a relevant application of aptamers to CAF targeting having not yet been described, the exploration of these molecules shows great potential. In this review, we provide an overview of the recent advances in RNA therapeutics for CAF targeting, discussing key examples as well as the main challenges and perspectives in the field.

## 2. Role of CAFs in Tumor Development and Progression

**Figure 1:**
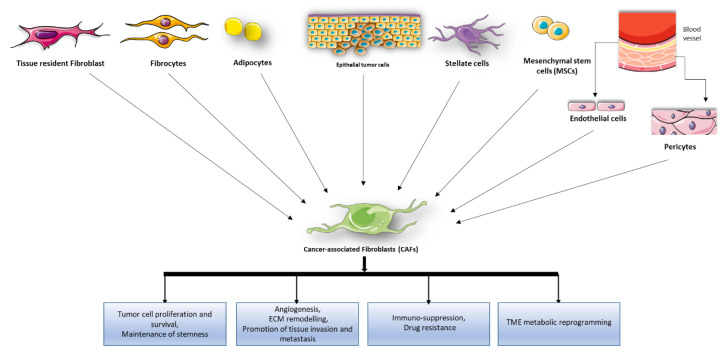
Cancer associated fibroblast (CAF) origins and pro-tumor functions. Potential cellular sources of CAFs include pre-existing resident fibroblasts and stellate cells, epithelial cells, endothelial cells, mesenchymal stem cells, circulating fibrocytes, pericytes, smooth muscle cells, and adipocytes. Activated CAFs exhibit enhanced proliferative and migratory properties and produce components of the extracellular matrix (ECM), growth factors and cytokines promoting ECM remodeling, angiogenesis, tumor cell survival, proliferation, tumor cell stemness maintenance, recruitment of immunosuppressive cells into the TME to assist in immune evasion, drug resistance, and tumor microenvironment (TME) metabolic reprogramming.

CAFs are activated mesenchymal cells, representing the most abundant population of stromal cells within the TME [5,15]. They are present in almost all the solid tumors in different proportions [16]. They are a heterogeneous cell population, that can originate from diverse cell types [5,17] through different stimuli, including local hypoxia, oxidative stress, and growth factors and cytokines, such as transforming growth factor beta (TGFβ), released from the neighboring tumor cells and infiltrating immune cells [18,19]. Interestingly, CAFs interact with tumor cells in a reciprocal manner and have been involved in tumor development at each stage [20] (Figure 1).

CAFs produce autocrine and/or paracrine growth factors and cytokines, which in turn promote main biological features of tumors, such as their migratory and invasive properties, growth, and survival. Among others, the production of TGFβ, leukemia inhibitory factor, growth arrest-specific protein 6, fibroblast growth factors (FGFs), growth differentiation factor 15 (GDF15), vascular endothelial growth factor (VEGF), platelet derived growth factors (PDGFs), and hepatocyte growth factor (HGF) has been reported [21].

In addition, widely studied CAF-derived proinflammatory cytokines influencing tumor growth and invasion include the CXC-chemokine ligand 12 (CXCL12). It is the ligand of CXCR4 expressed on cancer cells and plays an important regulatory role in both cancer initiation, angiogenesis, and metastasis [22]. It has been demonstrated that CXCL12 is able to induce angiogenesis by recruiting endothelial progenitor cells in breast cancer (BC), thereby providing sufficient nutrients to guarantee tumor growth and metastasis in vivo [23]. Notably, when mice bearing BC were treated with antibodies targeting CXCL12, reduced tumor volume and cell number were observed. Further, it has been shown that CAF-derived CXCL12 may function as an important EMT inducer BC cells by regulating the Wnt/β-catenin signaling pathway, thus promoting tumor cell migration and invasion. The involvement of CXCL12/CXCR4 signaling in tumor invasion and metastasis was also confirmed in other several cancer types, including non-small cell lung cancer (NSCLC), gastric cancer (GC) [24,25], and prostatic carcinoma (PCa) [26], and also linked to the promotion of cancer stem cell proliferation [27,28]. In addition to chemokines, CAFs also release several cytokines from the interleukin (IL) family, such as IL-6, able to promote the invasion of cancer cells into the surrounding matrix [29,30].

Besides, CAFs increase the invasiveness of cancer cells through the modification of cell–cell interactions and the re-modelling of the adjacent ECM. Indeed, they are a key source of ECM proteins, such as fibronectin and type I collagen, and actively modify the stromal ECM by enhancing matrix metalloproteinases (MMPs), including MMP9 and MMP13. These effects combined with CAF ability to control Rho/RAB mediated adhesion pathways, generate a permissive track in the stroma and help tumor cells to move to other sites [31].

The exchange of metabolites between cancer cells and the surrounding CAFs is an additional way of communication that can induce specific programs of cancer cell differentiation or metabolic switches further influencing tumor progression [32].

In recent years, extracellular endosome-derived vesicles called exosomes are also attracting growing interest as mediators of the crosstalk between CAFs and cancer cells. Exosomes are vesicles with a diameter of 50–150 nm, secreted by cells into circulation and containing nucleic acids and proteins. Several studies are showing that CAF-secreted exosomes can alter tumor phenotype and that, vice versa, cancer-derived exosomes can activate CAFs [33].

Concerning other pro-tumor functions of CAFs, their secretome and changes in ECM organization strongly influence cancer therapeutic response resulting in a protective effect that allows cancer cells to evade common therapies. In addition to limit drug penetration, CAF-remodeled ECM determines the gain of adhesive properties of cancer cells and induces EMT leading to therapeutic resistance [34,35]. This occurs by determining cell cycle arrest or by altering the expression of cellular transporters for drug uptake [36]. Moreover, CAFs may confer resistance to anticancer drugs through their soluble factors such as TGFβ, IL-6, and HGF [5].

Another important aspect is the participation of stromal cells in creating the unique cancer environment of chronic inflammation and immune tolerance. Stromal cells are a source of cytokines and chemokines which have both positive and negative effects on immuno responses [37]. However, CAFs predominantly sustain immunosuppressive processes. For example, key roles in reducing T-cell response have been demonstrated for IL-6, CXCL9, and TGFβ. As mentioned, CAFs excessively secrete MMPs which degrade basement membrane, and cleaved products of MMPs are chemotactic for leukocytes and modulate the proliferation of the immune cells. In addition, it has been recently observed that CAFs can cross-present antigens leading to the activation of CD4+ T cells and the suppression of CD8+ T cells.

All these studies highlight the great potential of therapeutic anti-cancer strategies targeting CAFs. However, it should be considered that the biology of CAF population remains still poorly defined and that despite CAF predominant functions are pro-tumorigenic, a higher complexity is recently emerging, suggesting a controversial tumor-suppressive role of some CAF “subtypes” [38].

## 3. Role of Endogenous ncRNAs in CAFs

### 3.1. MicroRNAs in CAFs

**Figure 2:**
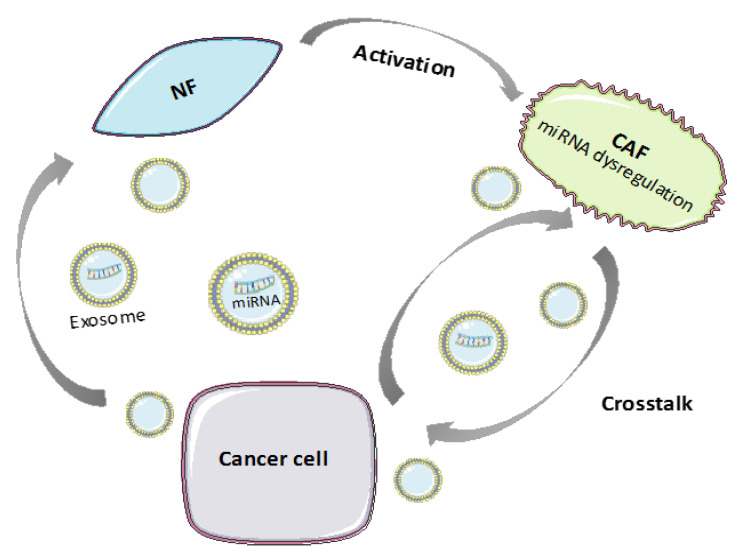
miRNAs play key role in the communication among normal fibroblasts (NFs), cancer associated fibroblast (CAFs), and cancer cells.

MicroRNAs are a class of small (17–25 nt) endogenous ncRNAs that regulate gene expression at a post-transcriptional level, both by messenger RNA degradation and protein translation suppression. miRs have the ability to regulate hundreds of target genes simultaneously thereby controlling multiple signaling pathways. As such, they have been shown to regulate every aspect of cellular activity, including differentiation and development, metabolism, proliferation and apoptosis [39,40]. They play a crucial role in human cancer development and tumorigenesis as their expression is frequently deregulated in many tumor types, functioning either as tumor suppressors or as oncogenes (oncomiRs). So far, increasing evidence indicates that miRNAs are involved in the transformation from NFs into CAFs and contribute to CAF-mediated cancer progression, metastasis, and chemoresistance (Figure 2) [41,42]. This section provides some key examples (summarized in Table 1).

#### 3.1.1. miRNA Deregulation in CAF Activation and Tumor-Promoting Functions

Several studies have focused on the differential miRNA expression pattern between CAFs and NFs to determine the key miRs contributing to the transformation of CAFs and to their tumor-promoting ability [74].

In one of the first examples, miRNA microarrays were used to analyze established primary cultures of CAFs and paired NFs from six resected BC tissues and served to identified 11 dysregulated miRNAs in CAFs: Three were up-regulated (miR-221-5p, miR-31-3p, miR-221-3p), while eight were down-regulated (miR-205, miR-200b, miR-200c, miR-141, miR-101, miR-342-3p, let-7g, miR-26b). Their target genes are known to affect cell differentiation, adhesion, migration, proliferation, secretion, and cell–cell interaction, indicating that the differentially expressed miRNAs might be involved in the tumor-promoting function of CAFs in BC [43]. A similar study was conducted on bladder cancer, concluding that the differentially expressed miRNAs (upregulation of miR-16 and miR-320, and downregulation of miR-243, miR-145 and miR-130) influence crucial cell processes and are involved in tumor development and progression of urinary bladder cancer [45].

Other studies, focusing on miRNA differential expression among CAFs and NFs, have exposed different functions of miRNAs in fibroblast reprogramming. Mitra et al. found that in ovarian CAFs, miR-31 and miR-214 were downregulated while miR-155 was upregulated when compared to normal or tumor-adjacent fibroblasts. Mimicking this deregulation by transfecting miRs and miR inhibitors induced a functional conversion of NFs into CAFs. This results in a large number of upregulated genes, highly enriched in chemokines, which are known to be important for CAF function. The most up-regulated chemokine, CCL5, was found to be a direct target of miR-214 [41].

A representative widely studied oncomiR is miR-21. It is up-regulated in various cancers and as such, its role in CAFs has begun to attract much attention. First notions of its role in fibroblast activation came from a couple of studies on primary cultured fibroblast that showed an induced upregulation of miR-21 after exposure to the TGFβ-1 and culture media from cancer cells [46,47]. In colorectal cancer (CRC), miR-21 overexpression was shown to drive the fibroblast-to-myofibroblast transdifferentiation through the upregulation of α-SMA [48]. More recently the effect of miR-21 has been studied in lung adenocarcinoma, hepatocellular carcinoma (HCC) and pancreatic cancer. Kunita et al. reported that conditioned medium from A549 lung cancer cells increased miR-21 expression in MRC-5 and IMR-90 lung fibroblasts through the TGFβ pathway, and induced CAF-like morphology and migratory capacity [49]. Further, miR-21 was associated to the metabolic alteration of CAFs and the effect of this alteration on pancreatic cancer cells. The authors demonstrated that compared with NFs, CAFs showed enhanced glucose uptake capacity, lactic acid production, and elevated miR-21 expression, and that after indirect co-culture with CAFs, oxidative phosphorylation and invasion ability of the pancreatic cancer cells was increased [50].

In PCa, studies on the molecular mechanisms leading to the activation of CAFs from tissue-resident fibroblasts have undercover several miRNAs, whose expression in normal prostate fibroblasts led to their conversion into CAF-like cells able to promote tumor induction and EMT. Among them, there are miR-409 [51], hypoxia-induced miR-210 [52], and muscle-specific miR-133b [53]. In addition to this upregulated miRs, Musumeci et al. demonstrated that miR-15 and miR-16 were downregulated in fibroblasts surrounding prostate tumor, and that such downregulation in CAFs promoted tumor growth and progression through the reduced post-transcriptional repression of *FGF-2* and its receptor *FGFR1*, which act on both stromal and tumor cells to enhance cancer cell survival, proliferation, and migration [54].

The role of CAFs’ miRs in GC has as well been documented in the latest years. Yang et al. investigated the differential expression of miRs in CAFs obtained from GC tissues versus matched normal gastric mucosa fibroblasts, revealing that miR-106b was significantly upregulated, a fact that was related to enhancing the proliferation, migration and invasion of tumor cells. Tumor suppressor gene, *PTEN*, was identified as a direct target of miR-106b [55]. Li at al. then identified miR-149 as a critical factor for the transformation of NFs into CAFs. MiR-149 expression levels were markedly lower in GC CAFs than in NFs, corresponding with higher secretion of its identified target, IL-6. Through the regulation of IL-6 expression, low levels of miR-149 promote the pro-tumor activity of CAFs by inducing EMT and stem-like traits in GC cells [56]. In another study, it was discovered that miR-214 was significantly downregulated in CAFs of GC compared with NFs, accelerating migration and invasion capabilities of GC cells by the EMT process. Secretory growth factor *FGF9* was proved to be a direct target gene of miR-214 [75].

Among dowregulated miRs, analyses in human CAFs isolated from endometrial cancer and normal endometrial fibroblasts identified miR-31 as being the most downregulated. Homeobox gene *SATB2* was demonstrated to be its direct target, whose expression was significantly elevated in CAFs and correlated with the expression of a number of genes involved in cell invasion, migration, and scattering [57]. The same authors discovered later the down-regulation of miR-148a in CAFs from endometrial cancer and confirmed WNT10B as a direct target gene of miR-148a [58].

In subsequent years, Yang et al. tried to unravel the role of miR-31 in CRC-associated fibroblasts. In this study, they conversely found that the expression of miR-31 in CAFs was higher than in NFs and demonstrated that miR-31 can inhibit autophagy in CAFs and increase the radiosensitivity of CRC, hypothesizing miR-31 as a new target for CRC treatments [59]. Similarly, Shen et al. revealed that miR-31 was up-regulated in lung CAFs. The authors also found miR-1 and miR-206 down-regulated. Importantly, modifying the expression of these three deregulated miRNAs induced a functional conversion of NFs into CAFs and promote the migration, colony formation, and tumor growth, as well as recruitment of tumor-associated macrophages (TAMs). *VEGFA*, *CCL2*, and *FOXO3a* were identified as direct targets of miR-1, miR-206, and miR-31, respectively [60].

Noteworthy, a cluster of miRs well characterized in CAFs is miR-200 family, including miR-200a, miR-200b, miR-200c, and miR-141. Tang et al. demonstrated that the miR-200 family were often downregulated in CAFs compared with paired NFs derived from BC patients. The downregulation of miR-200s can induce CAF-like characteristics in NFs in terms of increased expression of the CAF markers α-SMA and FAP and enhanced migration and invasion activity, contributing to enhance ECM remodeling and metastasis of BC [44].

In addition to tumor-promotion, most studies proposed CAFs to be closely associated with cancer chemoresistance and dysregulation of miRs in CAFs were involved in this function. For example, Tanaka et al. examined the role of extracellular miRs in the response to chemotherapy in esophageal cancer and discovered that high expression levels of miR-27a/b correlated with poor response to chemotherapy. Furthermore, MiR-27a/b transfected normal fibroblast showed α-SMA expression and increased production of TGF-β, indicating that miR-27a/b is involved in resistance to chemotherapy through the transformation of NFs into CAFs [61].

#### 3.1.2. MiRNA Transfer between CAFs and Tumor Cells through Extracellular Vesicles

More recently, increasing evidences suggest that miRs may be transferred between cells through extracellular vesicles, including exosomes.

A study aiming to identify and characterize exosomal miRs as key effectors of the communication between stroma and tumor cells in CRC, demonstrated that fibroblast exosomes are transferred to CRC cells, with a resultant increase in cellular miRNA levels, cancer proliferation and chemoresistance. An exosomal cancer-associated fibroblast signature consisting of miRNAs 329, 181a, 199b, 382, 215, and 21 was identified, being miR-21 the most abundant. Orthotopic xenografts established with miR-21-overexpressing fibroblasts and CRC cells led to increased liver metastases compared to those established with control fibroblasts [62].

The role of CAF exosomes and their miRs in the induction of the stemness and EMT phenotype of cancer cells have been documented in BC. Three miRs (miRs -21, -378e, and -143) were found to be overexpressed in exosomes from CAFs as compared from NFs, according to the study conducted by Donnarumma et al. They proved that miRs were transferred from CAFs to BC cells via exosomes, increasing mammosphere formation, stem cell and EMT markers, and anchorage-independent cell growth [63]. A very recent study proving as well that CAF-derived exosomes can transfer miRs in BC was conducted by Wang et al. They demonstrated the ability of miR-181d-5p to enhance the aggressiveness of BC through targeting caudal-related homeobox 2 (*CDX2*), a transcription factor binding to the promoter of homeobox A5 (HOXA5), whose overexpression is known to retard BC cell proliferation, invasion, migration, EMT and apoptosis. Effect of the downregulation of these two genes was proved in vivo showing that CAF-derived exosomes containing miR-181d-5p promoted the tumor growth in mice xenografts of MCF-7 cells [64].

In addition, Sun et al. discovered a new mechanism of CAF facilitated oral squamous cell carcinoma (OSCC) progression through exosomal release of miR-382-5p, pointing out that its overexpression was responsible for OSCC cell migration and invasion. Interestingly, CAF density in tumor tissues was found to be relevant to OSCC lymph node metastasis and tumor node metastasis stage [66].

A key role of exosomal miR transfer between CAFs and cancer cells was also associated to chemoresistance. Yeung et al. identified significantly higher levels of miR-21 in exosomes and tissue lysates isolated from CAFs than in those from ovarian cancer cells and demonstrated that miR-21 transferred from CAFs to cancer cells suppressed ovarian cancer apoptosis and conferred chemoresistance. This occurred by miR-21 regulation of the apoptotic protease activating factor 1 [67].

Concerning tumor drug response, it has been shown that CAFs exposed to chemotherapy play an active role in regulating the survival and proliferation of cancer cells. Richards et al. found that CAFs are intrinsically resistant to gemcitabine (GEM), the chemotherapeutic standard of care for pancreatic ductal adenocarcinoma. Further, they discovered that CAFs exposed to GEM prolifically secrete exosomes that contain chemoresistance-promoting factors like miR-146a, along with mRNA of its upstream transcription factor, Snail, which promotes chemoresistance, EMT, and metastasis [68]. In a later study Fang et al. found out that miR-106b level was as well upregulated in CAFs and CAFs-exosomes following GEM treatment and demonstrated its role in causing GEM resistance of pancreatic cancer [69].

Cisplatin resistance is a major drawback for advanced head and neck cancer (HNC) treatment. Under such premise, X. Qin et al. recently discovered a role for CAFs in regulating HNC cell survival and proliferation by delivering functional miR-196a from CAFs to tumor cells via exosomes. Exosomal miR-196a then binds novel targets, *CDKN1B* and *ING5*, to endow HNC cells with cisplatin resistance. Moreover, they also found that high levels of plasma exosomal miR-196a were clinically correlated with poor overall survival and chemoresistance [70]. Further, J. Hu et al. showed that CAFs contribute to growth, invasion, metastasis, and therapy resistance of human CRC by the transfer of CAF secreted exosomes to CRC cells, leading to a significant increase of miR-92a-3p. Mechanically, increased expression of miR-92a-3p activates Wnt/β-catenin pathway and inhibits mitochondrial apoptosis, contributing to cell stemness, EMT, metastasis, and 5-FU/L-OHP resistance [71].

Collectively these studies demonstrate how the transfer of miRs from CAFs to tumor cells participates in many CAF-promoting functions. Interestingly, it has been demonstrated that cancer cells can in turn use the same mechanism to communicate with the surrounding stroma inducing its transformation and initiating a “feed forward loop” where transformed stromal cells further sustain the tumors. For example, Zhou et al. showed that HCC cells exhibited a great capacity to convert normal hepatic stellate cells (HSCs) to CAFs. Crosstalk between tumor cells and HSCs was mediated by tumor-derived exosomes carrying miR-21 that directly targeted PTEN, leading to activation of PDK1/AKT signaling in HSCs. Activated CAFs further promoted tumor development by secreting different angiogenic cytokines [72]. A similar study was carried out by Fang et al. They reported that high-metastatic HCC cells secrete exosomal miR-1247-3p that directly targets *B4GALT3*, leading to activation of β1-integrin–NF-κB signaling in fibroblasts. Activated CAFs promote cancer progression by secreting pro-inflammatory cytokines, including IL-6 and IL-8. [73]. Further, another recent report regarding the development of CAFs from NFs in BC, showed that the transfer of miR-125b in EVs secreted from BC cells was responsible for fibroblast activation [65].

Summing up (Table 1), the above data support the idea that miRs mediate the crosstalk between tumor cells and CAFs, promoting and sustaining the progression of many solid tumors. All these evidences underline the great potential of using miRNA mimicking or miRNA inhibitors in order to enhance or reduce miR levels, respectively, reverting CAF phenotype, thus improving the effectiveness of the anticancer strategies.

### 3.2. lncRNAs in CAFs

LncRNAs are a heterogeneous class of ncRNA transcripts with a length of more than 200 nucleotides. They are transcribed from large intergenic regions or overlapping protein-coding genes and modulate different biological processes through a wide range of mechanisms including epigenetic, transcriptional and post-transcriptional regulation, RNA splicing and editing [76]. So far, many lncRNAs have been involved in cancer by acting directly as tumor suppressors or oncogenes, or by altering tumor suppressors and oncogenes [77]. More recently, it is emerging a significant role of lncRNAs in the regulation of TME components as well, including CAFs [78] (Table 2).

An essential role of lncRNAs as mediators of CAF pro-tumor factor network has been demonstrated. Zhuang et al. found that ZEB2NAT lncRNA is essential for the TGFβ-1-dependent processes involved in CAF-mediated induction of EMT and invasion of bladder cancer cells [79]. Similar results were found in BC by Ren Y et al. [80] that demonstrated that the TGF-β1 secreted by CAFs promotes EMT and metastasis through the induction of lnc-HOTAIR expression. Further, Zhang et al. [81] found that CAFs contribute to enhance the levels of midkine (MK), a heparin-binding growth factor promoting cancer drug resistance. MK released by CAFs enhances cisplatin resistance via the induction of lncRNA ANRIL. Likewise, it has been shown that CAFs promote the expression of the lncRNA DNM3OS in esophageal cancer cells, conferring radioresistance by regulating DNA damage response [82].

For instance, lncRNAs have been directly implicated in CAF transformation. In OSCC, a novel lncRNA, named Lnc-CAF, was found up-regulated in CAFs. Lnc-CAF is able to regulate NF reprogramming to CAFs inducing IL-33 levels by preventing its p62-dependent autophagy-lysosome degradation [83]. In addition, in ovarian cancer, it has been demonstrated a double action of LINC00092 lncRNA [84]. Indeed, LINC00092 contributes to maintain CAF-phenotype and simultaneously, is upregulated and induces a glycolytic phenotype in ovarian cancer cells in response to the chemokine CXCL14 released by CAFs.

In addition to miRs, the therapeutic potential of lncRNA carried by exosomes secreted by CAFs is also appearing in literature. Ren et al. [85] showed that lncRNA-H19 is up-regulated in CAFs as compared to NFs and that the transfer of H19 included in CAF-derived exosomes can promote stemness and chemoresistance in CRC. The mechanism of this effect involves H19 sponge action on miR-141 that results in the activation of the β-catenin pathway.

Despite, the clinical relevance of lncRNAs in TME is still at its infancy, these studies indicated their potential as therapeutic targets to be inhibit by means of specific molecules, such as siRNAs and ASOs, in order to improve anti-cancer strategies.

## 4. Synthetic RNA-Based Therapeutic Approaches for CAF Targeting

### 4.1. SiRNA Therapeutics in CAFs

SiRNAs are a class of small synthetic double-stranded RNAs which can induce gene silencing through sequence-specific cleavage of perfectly complementary mRNA [86]. In the last decades, the potential of siRNA-based therapy in cancer treatment has determined a huge concentration of studies and led to promising results on the inhibition of cancer cell growth and migration and the enhancement of drug response [87,88,89,90,91,92]. Recently, many investigators have focused their efforts on the use of siRNA-based therapy to reprogram the TME and alter the crosstalk between stromal and cancer cells [93,94,95,96,97,98] (Table 3).

In a study conducted in 2014, Leung and colleagues observed that the CAF-derived microfibrillar-associated protein 5 (MFAP5) is a negative prognostic marker in ovarian cancer and that its silencing inhibits the FAK/CREB/TNNC1 signaling pathway and significantly decreases ovarian tumor growth and metastasis in vivo [94]. In another recent investigation, Yang et al. demonstrated that the concomitant knockdown of glutamine synthase in the stroma and of glutaminase in cancer cells in a high-grade serous ovarian adenocarcinoma orthotopic mouse model disturbs the metabolic cross talk between cancer cells and CAFs. This leads to tumor regression, reducing tumor weight, nodules, and metastasis [95]. Furthermore, Chan and colleagues observed that the silencing of the nuclear receptor genes overexpressed in CAFs (RARβ, PPARβ/δ, VDR, GR, or AR) reduced squamous cell carcinoma (SCC) proliferation, invasiveness, energy metabolism, ROS production, and chemoresistance [96].

Recently, the influence of CAFs on the immune microenvironment have been investigated. Ford and colleagues found out that CAF-mediated suppression was due to the specific exclusion of CD8+ T cells from tumors. Furthermore, the authors demonstrated that the knockdown of NOX4 expression in CAFs inhibits CAF-dependent immunotherapy resistance in several murine CAF-reach tumor models, by overcoming the CD8+ T cells exclusion [98]. Another recent investigation showed that the crosstalk between CAFs and GC cells via the TNF-α/IL-33/ ST2L signaling pathway participates in GC progression. The authors demonstrated that the silencing of ST2L expression in cancer cells or of IL-33 in CAFs inhibited peritoneal spreading and metastatic capacity of GC cells in nude mice [97].

### 4.2. ASOs in CAFs

ASOs are small nucleic acids (usually 15-18 mer) complementary to a specific target mRNA. The hybridization of the ASO with the mRNA results in mRNA cleavage by the RNAse H, an enzyme which cleaves the RNA in an RNA-DNA duplex. The mRNA is thus no longer available for protein translation. Different ASOs have been proposed for the treatment of different human cancers [99] representing the first ON drugs widely used in clinical trials.

Even if the use of ASOs to alter CAF phenotype has been poorly explored, some of them, inhibiting important mediators of CAF pro-tumor functions, could show a potentiality also in CAF targeting. Main examples are represented by ASOs against TGF-β2 that are in clinical trials for the treatment of recurrent high-grade glioma, advanced pancreatic carcinoma, and metastatic melanoma [100], ASOs targeting angiogenic factors, such as epidermal growth factor receptor, in clinical trials for the treatment of e recurrent or metastatic squamous cell carcinoma of the head and neck, and VEGF, in clinical trials for the treatment of Kaposi sarcoma, mesothelioma, and renal cancer [101] or ASOs against bFGF [102] that are under preclinical investigations.

### 4.3. mRNAs Therapeutics in CAFs

mRNA therapeutics are a very promising class of drugs for the treatment of various pathologies, such as infectious diseases and cancer. However, for a long time their application has been limited by their instability and inefficient in vivo delivery. In recent years, advanced knowledge about the chemical modifications useful to improve mRNA pharmacology and the development of numerous nanomaterials aimed at protecting mRNAs from extracellular degradation and facilitating cellular uptake and endosomal escape have given an important boost to the field of mRNA-based anticancer vaccines [11]. Indeed, these approaches have demonstrated encouraging results in both animal models and humans. Currently, many clinical trials focused on the use of mRNA therapeutics for cancer treatment are ongoing. Some examples include the mRNA coding for the Wilms’ tumor protein (WT1) in clinical trial for the treatment of acute and chronic myeloid leukemia, multiple myeloma, mesothelioma, and glioblastoma; the mRNA coding for CD40L in clinical trial for the treatment of renal and pancreatic cancer; and a brain tumor stem cells-specific mRNA for the treatment of glioblastoma. Such an approach shows a great potentiality and may represent a valid system for CAF targeting in order to improve the efficacy of anticancer therapies [103].

### 4.4. Nucleic Acid-Based Aptamers in CAFs

**Figure 3:**
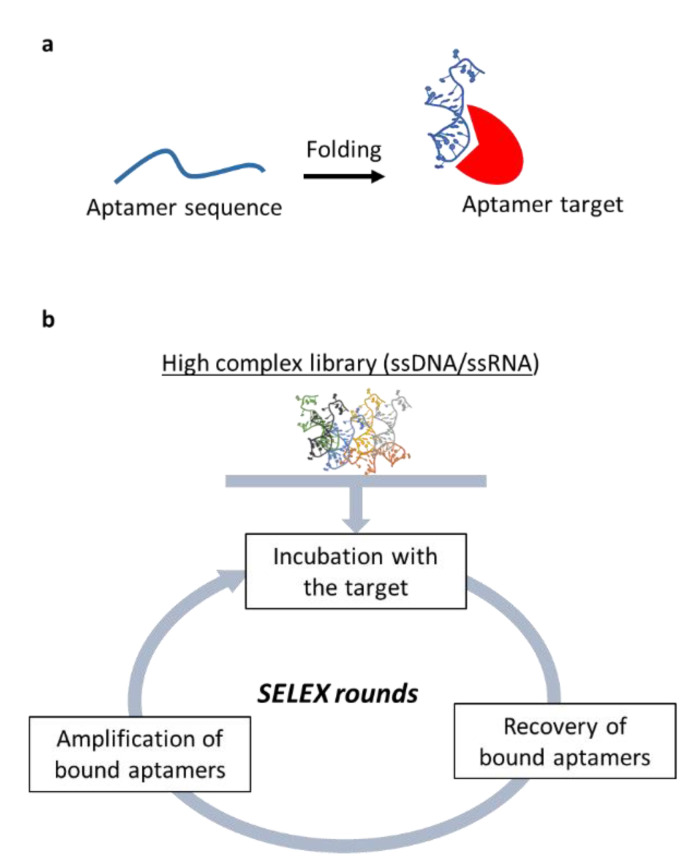
Nucleic acid-based aptamers. (**a**) Schematic representation of aptamer-target binding that depends on aptamer folding. (**b**) The SELEX (Systematic Evolution of Ligands by EXponential enrichment) process permits to select aptamers from high complex library of a random oligonucleotides through repeated rounds of binding, partitioning, and amplification of bound oligonucleotides.

Nucleic-acid aptamers are short single-stranded oligonucleotides (of DNA or RNA) that bind their target by folding in specific 3D structures (Figure 3a). Therefore, aptamer mode of action is comparable to that of monoclonal antibodies to which they are often likened. Aptamers show low affinity as antibodies and possess many advantages including superior specificity and stability, easier production and cost-effectiveness. In addition, no immunogenicity has been reported for aptamers and their chemistry is suitable for easy modification to allow their labeling or to improve their stability and pharmacokinetic [104]. Aptamers are selected in vitro by a combinatorial chemistry approach named SELEX (Systematic Evolution of Ligands by EXponential enrichment) (Figure 3b) that has been applied to many different targets [105], leading to the isolation of a wide range of aptamers against therapeutically relevant targets in human pathologies including cancer [106]. Thanks to their unique features, aptamers offer exquisite tools to: 1) Specifically recognize cancer epitopes; 3) act as inhibitor to antagonize the malignant phenotype; and 4) act as delivery carriers for cancer therapeutics allowing their specific internalization within the target cells thus reducing unwanted off target effects [107].

So far, within the TME, aptamers have been mostly applied to the development of innovative immunotherapeutic strategies redirecting the immune system cells and favoring the anti-tumor immune-mediated responses [108]. On the contrary, the use of aptamers to alter CAF phenotype has been poorly explored up to date. In this regard, an interesting study has been recently reported by Camorani et al. [109]. They demonstrated that a previously characterized aptamer binding and inhibiting the PDGF receptor beta [110] blocks bone marrow-derived mesenchymal stem cells trans-differentiation into CAFs reducing their recruitment into TME and their pro-metastatic action in triple negative BC. This study underlines how exploring aptamers to target CAF phenotype shows great potential to open innovative horizons in the current therapeutic approaches.

Further, since it has been demonstrated that aptamers against cell surface receptors may specifically internalize within the target cells, the use of aptamers as delivery carriers has emerged powerfully in the last decades. One main limit for the development of RNA therapeutics for cancer treatment is represented by the difficulty in delivering them to the site of interest because of the presence in the organism of several functional and physical obstacles impeding their penetration into target cells following systemic administration. Thus, the use of ligands enabling active binding and internalization represents an attractive strategy and a key step toward their clinical development. In this context, the high affinity and specificity for their targets, combined to their easily modifiable chemical nature, have supported the development of different aptamer-based conjugates for the targeted delivery of several secondary reagents, including RNA therapeutics [111]. It has been widely demonstrated that aptamers can allow the selective receptor-dependent RNA internalization within the target cells, thus avoiding the occurrence of unwanted off-target effects [112].

## 5. Conclusions

The development of strategies aimed to specifically target CAF pro-tumor function should enhance the effectiveness of the therapeutic interventions. In this view, the selective CAF targeting represent a great challenge in oncology and nucleic acid therapeutics may provide promising molecules. There is a growing effort in characterizing the role of ncRNAs in the regulation of CAFs and uncovering novel master gene regulators that can guide the design of siRNA-based therapeutic tools. All these RNA-based therapeutics are very promising, however, there are some issues to be resolved to improve their use in clinical trials, such as the effective intracellular delivery to the target site, improvement of their stability and reduction of non-specific toxicity [113,114]. In particular, a major challenge is represented by their appropriate delivery to target cells, comprising cellular uptake across the lipid bilayer, endosomal escape, and localization. To overcome these limitations, RNA-based therapeutics can be delivered via nonspecific uptake using nano-sized carriers, such as polymeric nanoparticles, lipid nanoparticles, mesoporous silica nanoparticles, gold and magnetic nanoparticles, and single-walled carbon nanotubes. These intracellular delivery systems would enable an encapsulation of the loading cargo and its protection from degradation. An alternative to the use of nanoparticles is to directly conjugate a bioactive ligand to the RNA, allowing for a receptor-mediated uptake. Examples of this technique includes the conjugation of N-acetylgalactosamine (GalNAc) for liver targeting, as well as the use of specific antibodies and aptamers [115,116]. By taking advantage of their high affinity and specificity and their ability to be effectively internalized into cells, a variety of aptamers specific to cancer biomarkers have been conjugated with therapeutic RNA agents as siRNAs and miRs have proved to be valid alternatives for targeted delivery in a cell type-specific manner [117], without inducing undesired toxic effects in the organism. In addition, CAFs are heterogeneous populations and the detailed characterization of their profiles is still a challenging point towards the development of effective molecules for their therapeutic targeting.

## Figures and Tables

**Table 1:** Most relevant miRNAs implied in CAFs activation and their tumor prompting functions.

Operating Mode	MiRNA	De-Regulation Direction	Mechanism of Action	Cancer Type
miRNA deregulation in CAF activation and tumor-promoting functions	miR-221-5p, miR-31-3p, miR-221-3p	Up-regulation	Differentiation, adhesion, migration, proliferation, and cell–cell interaction	Breast cancer [43,44]
	miR-205, miR-200b, miR-200c, miR-141, miR-101, miR-342-3p, let-7g, miR-26b	Down-regulation		
	miRs-200 family, miR-141	Down-regulation	Conversion of normal fibroblasts (NFs) into CAFs	
	miR-16, miR-320	Up-regulation	Tumor development and progression	Bladder cancer [45]
	miR-243, miR-145 and miR-130	Down-regulation		
	miR-155	Up-regulation	Conversion of NFs into CAFs	Ovarian cancer [41]
	miR-31, miR-214	Down-regulation		
	miR-21	Up-regulation	CAF activation	Colorectal cancer, lung adenocarcinoma, hepatocellular carcinoma [46,47,48,49]
		Up-regulation	Metabolic alterations of CAFs	Pancreatic cancer [50]
	miR-409, miR-210, miR-133b	Up-regulation	Conversion of NFs into CAFs Tumor induction and epithelial–mesenchymal transition (EMT)	Prostate carcinoma [51,52,53,54]
	miR-15, miR-16	Down-regulation	Tumor growth and progression	
	miR-106	Up-regulation	Proliferation, migration and invasion of tumor cells	Gastric cancer [55,56]
	miR-149	Down-regulation	Transformation of NFs into CAFs	
	miR-214	Down-regulation	EMT	
	miR-31	Down-regulation	Cell invasion, migration and scattering	Endometrial cancer [57,58]
	miR-148	Down-regulation	Activation of the WNT/b-catenin pathway	
	miR-31	Up-regulation	Cancer development	Colorectal cancer [59,60]
	miR-1, miR-206	Down-regulation	Conversion of NFs into CAFs Migration, colony formation, and tumor growth Recruitment of tumor-associated macrophages (TAMs)	
	miR-27a/b	Up-regulation	Poor response to chemotherapy	Esophageal cancer [61]
MiRNA transfer between CAFs and tumor cells through extracellular vesicles	miR-329, miR-181a, miR-199b, miR-382, miR-215, miR-21	Over-expression	Cancer proliferation and chemoresistance	Colorectal cancer [62]
	miR-21, miR-378e, miR-143		Induction of the stemness and EMT phenotype of cancer cells	Breast Cancer [63,64,65]
	miR-125b		Development of CAFs from NFs	
	miR-181d-5p		Cell proliferation, invasion, migration, EMT and apoptosis	
	miR-382-5p		Cell migration and invasion	Oral squamous cell carcinoma [66]
	miR-21		Chemoresistance	Ovarian cancer [67]
	miR-146a, miR-106b		Gemcitabine (GEM) resistance	Pancreatic cancer [68,69]
	miR-196a		Cisplatin resistance	Head and neck cancer [70]
	miR-92a-3p		Cell stemness, EMT, metastasis and 5-FU/L-OHP resistance	Colorectal cancer [71]
	miR-21		CAF activation	Hepatocellular carcinoma [72,73]
	miR-1247-3p			

**Table 2:** Examples of lncRNAs involved in the crosstalk between CAFs and tumor cells.

lncRNA	Mechanisms of Action	Cancer Type
ZEB2NAT	CAF-secreted TGFβ1 induces EMT and invasion via lncRNA-ZEB2NAT	Bladder cancer [79]
HOTAIR	CAF-secreted TGFβ1 induces EMT and metastasis via LncRNA-HOTAIR	Breast cancer [80]
ANRIL	MK released by CAFs enhances cisplatin resistance via the induction of lncRNA-ANRIL.	Oral squamous cell carcinoma [81]
DNM3OS	CAFs confer radioresistance promoting the expression lncRNA-DNM3OS via PDGFβ/PDGFRβ/FOXO1 signaling pathway	Esophageal cancer cells [82]
Lnc-CAF	Lnc-CAF increase IL-33 expression inducing CAF transformation	Oral squamous cell carcinoma [83]
LINC00092	LINC00092 maintains CAF-phenotype and is simultaneously induced in cancer cells by CAFs-secreted CXCL14 promoting cancer metastasis	Ovarian cancer [84]
H19	LncRNA-H19 carried by CAF-derived exosomes can promote stemness and chemoresistance	Colorectal cancer [85]

**Table 3:** Examples of small interfering RNAs (siRNAs) involved in the crosstalk between CAFs and tumor cells.

siRNA Target	Mechanisms of Action	Cancer Type
MFAP5	Silencing of MFAP5 expression in CAFs, inhibited ovarian tumor growth, invasion, and metastasis	high-grade serous ovarian cancer [94]
Stromal glutamine synthetase and cancer cell glutaminase	The simultaneous silencing of glutamine synthetase in the stroma and glutaminase in cancer cells, disrupts CAFs/cancer cells metabolic crosstalk, inducing tumor regression	high-grade serous ovarian adenocarcinoma [95]
RARβ, PPARβ/δ, VDR, GR and AR	knockdown in CAFs leads to attenuation of SCC invasiveness, proliferation, energy metabolism, ROS production and response to chemotherapy	Squamous cell carcinoma [96]
IL-33 ior ST2L	Targeting IL-33 expression in CAFs or ST2L expression in gastric cancer (GC) cells inhibits the metastatic capacity of GC cells in nude mice.	Gastric cancer [97]
NOX4	NOX4 inhibition suppresses CAF-mediated immunotherapy resistance	lung, colorectal and breast cancers [98]
